# LC–MS-Based Lipidomic Analysis of Serum Samples from Spontaneously Hypertensive Rats Treated with an Extract of *Acanthopanax sessiliflorus* Fruits

**DOI:** 10.3390/molecules25143269

**Published:** 2020-07-17

**Authors:** Dae Young Lee, Bo-Ram Choi, Dahye Yoon, Hyoung-Geun Kim, Min-Ho Lee, Geum-Soog Kim, Young-Seob Lee

**Affiliations:** 1 Department of Herbal Crop Research, National Institute of Horticultural and Herbal Science, RDA, Eumseong 27709, Korea; dylee0809@gmail.com (D.Y.L.); bmcbr@korea.kr (B.-R.C.); dahyeyoon@korea.kr (D.Y.); kimgs0725@korea.kr (G.-S.K.); 2 Graduate School of Biotechnology, Kyung Hee University, Yongin 17104, Korea; zwang05@khu.ac.kr; 3 Department of Food Technology and Services, Eulji University, Seongnam 13135, Korea; minho@eulji.ac.kr

**Keywords:** *Acanthopanax sessiliflorus* fruits, hypertension, LC/MS, lipidomics, serum

## Abstract

Recently, lipidomics has revealed that many diseases are highly associated with altered lipid metabolism, as in the case of hypertension affecting serum lipid metabolism. In this study, an LC–MS-based lipidomic approach was used to profile serum lipids in spontaneously hypertensive rats (SHRs) treated with an extract of *Acanthopanax sessiliflorus* fruits (ASF), to elucidate the serum lipid metabolism alteration by hypertension and the treatment of a drug or ASF. First, UPLC-QTOF/MS profiled a total of 208 lipids from six pooled samples of normal controls, SHR, SHR + 100 mg/kg of drug, and SHR + ASF 200, 400, or 600 mg/kg. These six groups were differentiated by the PCA and sPLS–DA, and 120 lipid species were identified as differentially regulated lipids (DRLs) by ANOVA (*p* values < 0.05). Second, UPLC–QqQ/MS was used for the target profiling of 120 DRLs from individual samples of the six groups. Using an ANOVA, 67 lipids (38 TGs, 4 DGs, 17 PCs, 2 PEs, and 6 LPCs) were selected as validated DRLs. The mostly altered lipids, such as TG (62:13), TG (60:13), PC (34:4), PC (36:5), and PC (38:2), were decreased in SHR compared to the normal control, and received little by treatment with ASF. These results demonstrated the correlation between hypertension and serum lipid metabolism. Furthermore, both drug and ASF treatment similarly altered the lipid profiles of SHRs. Finally, we found that DRLs have the potential to help us to interpret the lipid metabolism of hypertension.

## 1. Introduction

Hypertension is a clinical syndrome leading to many chronic diseases, such as cardiovascular disease (CVD), stroke, renal disease, and diabetes [1,2]. CVD is a major cause of morbidity and mortality worldwide, and there is significant global interest in the prevention and treatment of CVD [3]. Although hypertension is widely known as one of the main risk factors of CVD, the underlying mechanisms are not clearly understood. For the prevention of CVD, it is critical to research the mechanisms of the development of hypertension, as well as those of increased blood pressure. Traditionally, an atherogenic lipid profile with high levels of total cholesterol, low-density lipoprotein cholesterol, triglycerides, and low levels of high-density lipoprotein cholesterol was considered to be a risk factor for CVD [4]. Furthermore, high blood pressure, combined with undesirable levels of cholesterol can cause oxidative stress, vascular inflammatory responses, and atherosclerosis [5,6,7].

Abnormal lipid homeostasis in the blood is closely related to CVD and hypertension, and was recently studied in view of the altered composition of lipids in vivo [8]. Recently, it was reported that sphingolipids have a pathological role in hypertension. In human genetic analysis, it was found that sphingolipid metabolism is related to the regulation of blood pressure and hypertension [9]. Plasma lipidomics analysis of spontaneously hypertensive rats (SHR) has also shown that altered sphingolipid signaling is associated with a mechanism to prevent a rise in blood pressure [10]. Until now, sphingolipids were identified as hypertension-related lipids. However, it still remains unclear how whole lipids, like neutral lipids and phospholipids, are altered by hypertension. For detailed characterization of whole lipids that are differentially regulated by hypertension, a lipidomics approach based on liquid chromatography (LC) coupled with mass spectrometry (MS) is required.

Recently, ultra-performance LC (UPLC) coupled with tandem MS (MS/MS) was used to analyze various lipids from biological samples [11,12,13]. The UPLC system with a small particle size column provides high-throughput, high-resolution separation of various lipid species [14,15]. For comprehensive lipid profiling, the quadrupole time of flight (QTOF)/MS is an effective tool to fully mass scan with sensitivity, and achieves exact mass measurement of compounds [16,17]. Multiple reaction monitoring (MRM) based on triple quadrupole (QqQ)/MS is also effective for the sensitive and selective profiling of target compounds [18,19]. In this study, we used these analytical systems—UPLC–QTOF/MS and UPLC–QqQ/MS—to analyze various lipids in the serum of SHR. SHR is a good animal model for hypertension because its pathophysiological processes are similar to those of human beings [20]. Serum lipid profiling of SHR might provide a good understanding of hypertension-related lipid metabolism.

*Acanthopanax sessiliflorus* (Rupr. et Maxim.) is widely distributed in Northeast Asian countries, including Korea, China, and Japan. Its fruits were used to develop various food therapy products, as they are proven to be effective in cardiovascular diseases without toxicity [21,22,23]. As such, an ethanol extract of *A. sessiliflorus* fruits (ASF) has a significant antiplatelet activity [24]. The activation of platelets occurs in hypertension, and platelet aggregation is involved in the development of vascular complications related to hypertension [25]. Jung et al. [26] reported an improvement of vascular relaxation and decrease in blood pressure in the hypertensive animal model. Especially, 600 mg/kg of ASF decreased blood pressure to levels comparable to the captopril-administered group. In this study, we analyzed serum samples from the SHR model treated with ASF and positive control (captopril). The objective of this study was to identify major markers and characterize the process through which the rat serum lipid metabolism was altered by hypertension and treatment with a captopril (drug) and ASF, using comprehensive serum lipidome profiling, based on multiple reaction monitoring. Additionally, differentially regulated lipids (DRLs) identified from a serum lipidome profiling experiment were validated using MRM, a highly sensitive and reproducible verification platform that enables the quantification of a large number of candidate lipids in a single experiment with high throughput [27]. We further demonstrated a visualization of the general clustering trend plots from the serum samples, using multivariate statistical analysis.

## 2. Results and Discussion

### 2.1. Overall Workflow of the Study

**Figure 1:**
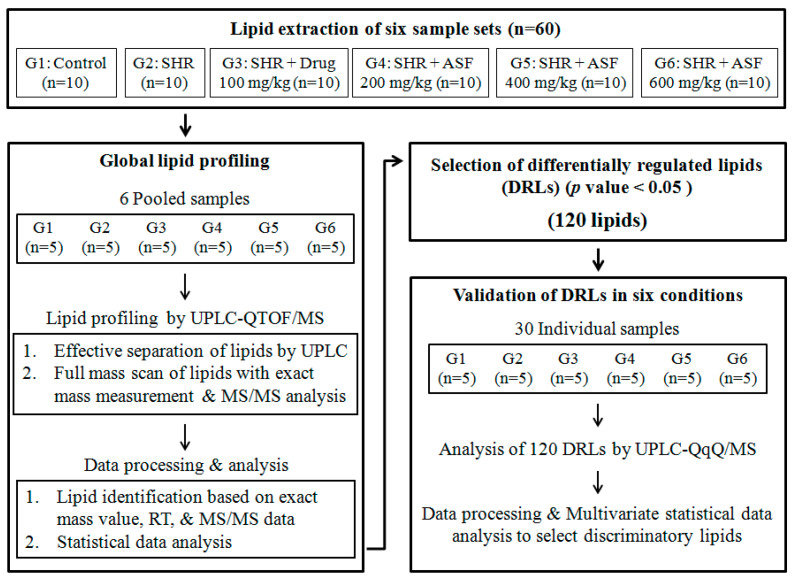
The overall scheme of the lipidomic analysis used to find the discriminatory lipids for six groups, including the normal control (G1), spontaneously hypertensive rats (SHR) (G2), SHR + Drug 100 mg/kg (G3), SHR + ASF 200 mg/kg (G4), SHR + ASF 400 mg/kg (G5), and SHR + ASF 600 mg/kg (G6).

We performed lipid profiling of rat serums obtained from six sample sets, including the normal control (n = 10), SHR (n = 10), SHR + Drug 100 mg/kg (n = 10), SHR + ASF 200 mg/kg (n = 10), SHR + ASF 400 mg/kg (n = 10), and SHR + ASF 600 mg/kg (n = 10). First, global lipid profiling of the pooled serum samples was performed to find the differentially regulated lipids (DRLs) of the six conditions. Lipid extraction of each serum sample was carried out. Then, five individual lipid extracts were mixed to prepare a pooled sample for a single condition. UPLC–QTOF/MS was used to profile lipids in the six pooled samples. Various lipids were well separated by UPLC, and the MSE acquisition mode based on QTOF/MS was used for the full mass scan of lipids with exact mass measurements and MS/MS analysis. In the data processing, various lipids were assigned on the basis of their exact mass values and RTs. The fatty acyl composition of various lipid species was also assigned using the MS/MS data. Second, the lipid profiles of the six conditions were subjected to statistical data analysis. The statistically different lipids among the six groups (*p*-value < 0.05) were selected as the DRLs of the normal control (G1), SHR (G2), SHR + Drug 100 mg/kg (G3), SHR + ASF 200 mg/kg (G4), SHR + ASF 400 mg/kg (G5), and SHR + ASF 600 mg/kg (G6). As a result, 120 lipids were selected as DRLs. Third, the DRLs were validated by individual sample analysis. The lipid extracts obtained from 30 individual samples, including G1 (n = 5), G2 (n = 5), G3 (n = 5), G4 (n = 5), G5 (n = 5), and G6 (n = 5), were subjected to target profiling of 120 DRL species, using UPLC–QqQ/MS. MRM was applied to the relative quantification of target lipids. In the data processing, the peak area of each compound was calculated and normalized by the peak area of IS. Finally, multivariate statistical analysis was applied to select the validated DRLs (vDRLs) for distinguishing each condition. The overall scheme is shown in Figure 1.

### 2.2. Global Lipid Profiling of Rat Serum by UPLC–QTOF/MS

**Figure 2:**
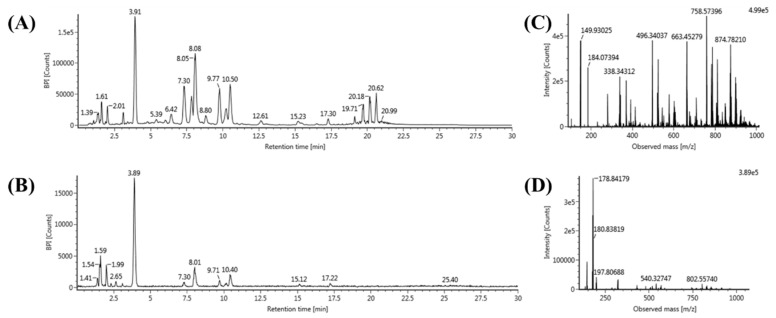
The base peak intensity (BPI) chromatogram of lipid extracts obtained from rat serum in the positive ion mode (**A**) and negative ion mode (**B**). Mass spectrum of lipid extracts obtained from rat serum in the positive ion mode (**C**) and negative ion mode (**D**).

For the profiling of various lipids, we applied a lipidomics platform based on UPLC–QTOF/MS that enabled fast and sensitive analysis with high mass accuracy. First, twelve lipid standards were used to optimize the UPLC–QTOF/MS conditions to analyze TG, DG, PC, PE, PG, PI, LPC, LPE, LPG, LPI, SM, and Cer. In the positive ion mode of ESI, each lipid was detected with several adducted ions, such as a hydrogen ion (H+), a sodium ion (Na+), and an ammonium ion (NH4+). For example, TG was detected as an [M+NH4]^+^ ion, and DG was detected as an [M+Na]^+^ ion. Furthermore, PC, PE, LPC, LPE, SM, and Cer were detected as [M+H]^+^ ions. In the negative mode, PG, PI, LPG, and LPI were detected as [M-H]- ions. Second, the validation of the lipid analysis using UPLC–QTOF/MS was performed to estimate its performance. Each lipid standard was analyzed six times to assess the repeatability of RT and peak intensity. The relative standard deviation (RSD) (%) of RT and peak intensity were smaller than 1.1% and 9.9%, respectively. The method showed high repeatability, and the correlation (R2) in each lipid analysis was at least 0.9909, indicating high reliability. The limits of detection (LODs) of each lipid standard are listed in Table S1. Next, we performed lipid extraction on 30 rat serum samples obtained from G1 (n = 5), G2 (n = 5), G3 (n = 5), G4 (n = 5), G5 (n = 5), and G6 (n = 5). Five individual lipid extracts per experimental group were mixed to prepare a pooled sample for one group. Lipid profiling of six pooled samples was performed to find the DRLs of the six groups. Each pooled sample was analyzed by triple replication (n = 3). For the positive and negative ion modes, the base peak intensity (BPI) chromatograms and MS spectrum of rat serum lipids are represented in Figure 2. As a result, the negative mode showed a few peaks in the chromatogram and MS spectrum, and various lipids were successfully analyzed in the positive mode. The lipid profiles of the six pooled samples were subjected to data processing using the UNIFI software (version 1.8; Waters Corporation). The information of various lipid species’ molecular formulas and RTs was applied to identify the analyzed lipids. The precursor ion m/z of the lipids obtained by exact mass measurement based on QTOF/MS was critical for lipid identification. Furthermore, the alternative high- and low-energy scans conducted by the MS^E^ mode represent the product ions that can be assigned to the structure of lipids. Finally, various lipid species (120 TGs, 12 DGs, 36 PCs, 13 PEs, 3 PIs, 17 LPCs, 5 LPEs, and 2 LPIs) were determined with RT and mass accuracy (ppm) (Table S2).

### 2.3. Statistical Data Analysis and Selection of DRLs of the Six Groups

The lipid datasets of the six pooled samples were subjected to statistical analysis. To visualize the general clustering trends among the six groups, we used multivariate analyses like principal component analysis (PCA) that indicated if any differences exist among the samples and the sparse partial least squares—discriminant analysis (sPLS–DA) that identified which components alter were applied for discriminating the six groups.

**Figure 3:**
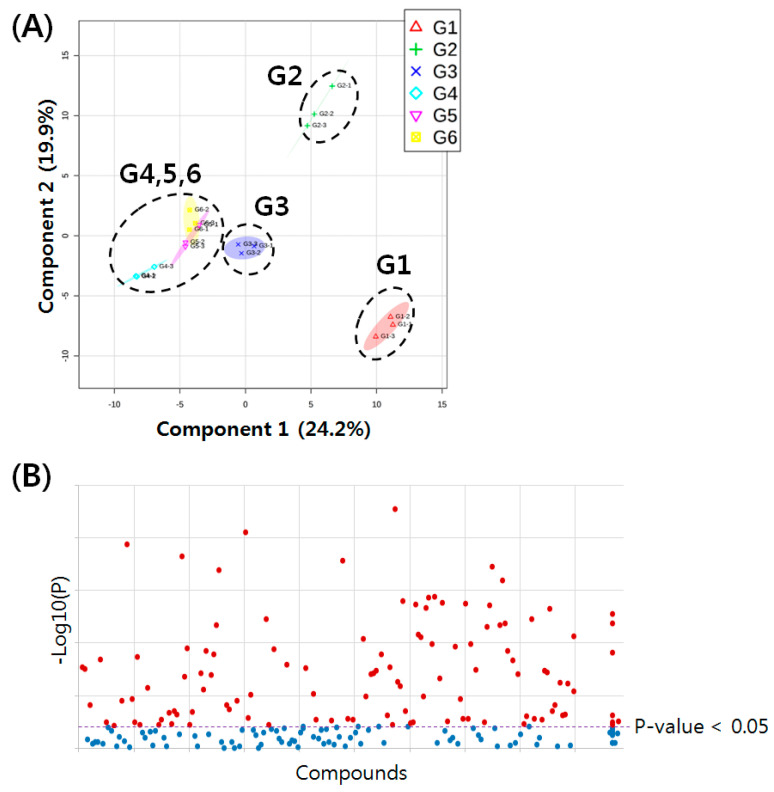
(**A**) sPLS–DA score plot of lipid profiles obtained from six pooled samples of the normal control (G1), SHR (G2), SHR + Drug 100 mg/kg (G3), SHR + ASF 200 mg/kg (G4), SHR + ASF 400 mg/kg (G5), and SHR + ASF 600 mg/kg (G6). (**B**) ANOVA representing the statistically altered lipids among the six pooled samples (G1-6) (*p* value < 0.05).

The score plots of PCA represent analyses that described 67.3% of the total variance, in which optimal segregation was achieved between principal component 1 (50.1%) and principal component 2 (17.2%) (Figure S1). Among the six groups, G1 and G2 were separated in the PCA score plot, although the triple replicate points of G2 were not clustered with each other. The other four groups were also plotted in the center of both G1 and G2. To better discriminate the six groups, we also applied sPLS-DA, which reduced the number of variables in the data to produce a robust and easy-to-interpret model. As a result, the sPLS–DA score plots described 44.1% of the total variance, in which optimal segregation was achieved between Component 1 (24.2%) and Component 2 (19.9%) (Figure 3A). G1, G2, and G3 were well separated, and the other groups, including G4, G5, and G6, were scattered on the left side of the plots of G3. This indicated that the rat serum lipid profiles differed, depending on the normal control, SHR, and SHR + Drug, and treatment with both the drug and ASF showed similar lipid profiles. To find the DRLs, which were the statistically different lipids among the six groups, we performed ANOVA tests (Figure 3B). Using the Student’s t-test, the *p* values of 120 lipids were all below 0.05. These lipids were selected as the DRLs of the six pooled samples, and are listed in Table S3.

### 2.4. Validation of Selected DRLs by the Target Profiling of the Six Groups

To validate the selected 120 lipids as the DRLs of the six groups, we performed target profiling of the 120 lipids in 30 individual samples. The 120 lipids were classified into six lipid classes, including TG, DG, PC, PE, LPC, and LPE. Thus, we used a previously constructed UPLC–QqQ/MS-based method for the target profiling of TG, DG, PC, PE, LPC, and LPE. Each individual sample was analyzed in triplicates (n = 3). In the data processing, the peak area of each lipid species in the MRM data was calculated using the Skyline software. The peak area of individual lipid species was then normalized by the peak area of the IS. For example, TG (11:1-11:1-11:1) was used to normalize individual TG species. Other standards, including DG (8:0-8:0), PC (10:0-10:0), PE (10:0-10:0), LPC (13:0), and LPE (14:0), were also used for the normalization of each lipid class.

**Figure 4:**
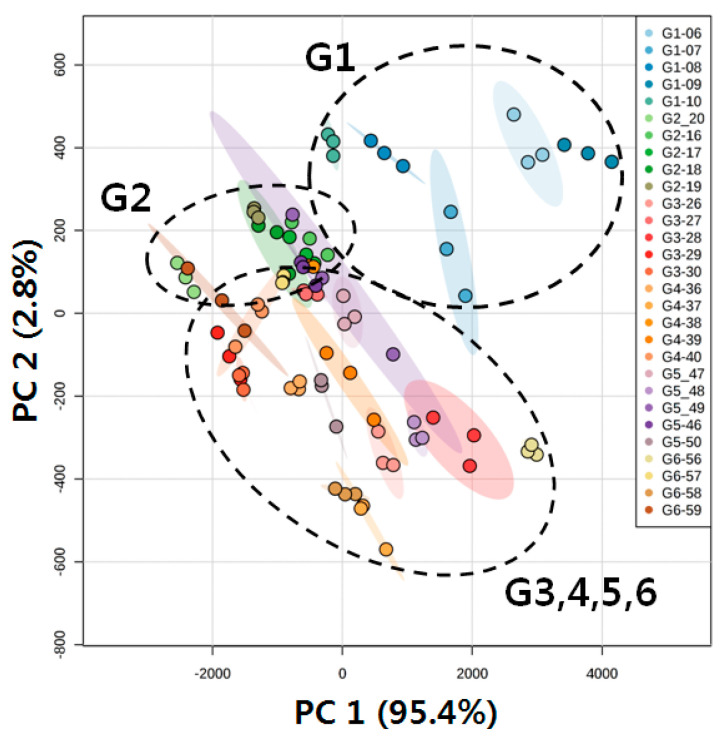
PCA score plot of lipid profiles obtained from thirty individual samples of normal controls (G1) (n = 5), SHR (G2) (n = 5), SHR + Drug 100 mg/kg (G3) (n = 5), SHR + ASF 200 mg/kg (G4) (n = 5), SHR + ASF 400 mg/kg (G5) (n = 5), and SHR + ASF 600 mg/kg (G6) (n = 5).

Next, the datasets of 120 DRLs from 30 individual samples were subjected to statistical analysis for the selection of vDRLs to distinguish the six groups. First, PCA was performed to visualize the general clustering trends of 30 individual plots from the six groups (Figure 4). The score plots of PCA represent analyses that described 98.2% of the total variance, in which optimal segregation was achieved between principal component 1 (95.4%) and principal component 2 (2.8%). This showed a higher value of total variance than the PCA score plots of the six pooled samples data. Both G1 (15 points) and G2 (15 points) were separated from each other in the score plot. Although the 60 points of G3, G4, G5, and G6 were not well separated from each other, these four groups were scattered on the lower side of the other two groups, G1 and G2. This indicated that the selected 120 lipids were differentially regulated in G1 and G2 compared to the other four groups. The pathological difference between the normal control and SHR seemed to correlate with their different lipid profiles. Furthermore, treatment with the drug or ASF altered the serum lipid profiles, curing hypertension. This demonstrated that hypertension and treatment with the drug or ASF had effects on the serum lipid metabolism.

**Figure 5:**
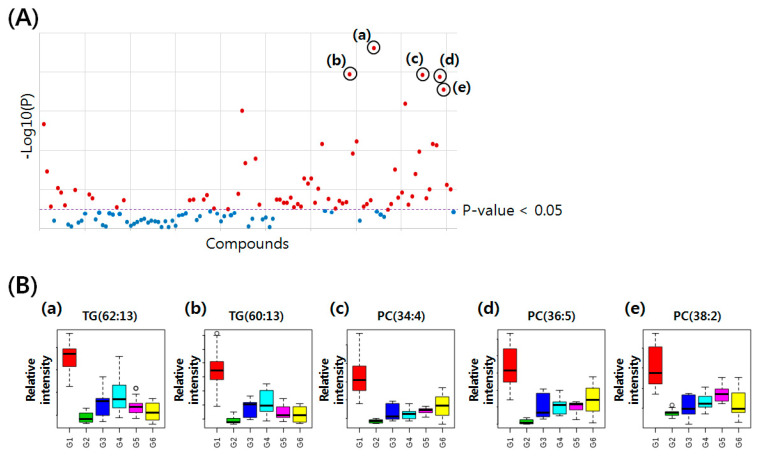
(**A**) ANOVA representing the statistically altered compounds among the 120 lipids from thirty individual samples of G1–6 (*p* value < 0.05). The most significantly altered five lipid species were—(**a**) TG (62:13), (**b**) TG (60:13), (**c**) PC (34:4), (**d**) PC (36:5), and (**e**) PC (38:2). (**B**) The bar plots show the relatively altered levels of these five lipids in the six groups.

An ANOVA test was applied to select vDRLs for the six groups (Figure 5A). Using the Student’s t-test (*p* value < 0.05), 67 lipids (38 TGs, 4 DGs, 17 PCs, 2 PEs, and 6 LPCs) were selected as the vDRLs (Table 1). Among the 67 vDRLs, the most significantly altered five lipid species were TG (62:13), TG (60:13), PC (34:4), PC (36:5), and PC (38:2). In Figure 5B, the bar plots show the relatively altered levels of these lipids according to the six groups. Interestingly, these lipids were decreased in SHR, compared to the normal controls. Furthermore, compared to SHR, these lipids were increased a little in the SHR with treatment by a drug or ASF. This indicated that the levels of several lipids in the rat serum were correlated with hypertension and drug or ASF treatment.

## 3. Materials and Methods

### 3.1. Standard Constituents and Reagents

HPLC-grade methanol, acetonitrile, water, and 2-propanol were purchased from J.T. Baker (Avantor Performance Material, Center Valley, PA, USA). Chloroform and ammonium formate were purchased from Sigma-Aldrich (St. Louis, MO, USA). HPLC-grade formic acid was purchased from Fluka Analytical, Sigma Aldrich Chemie GmbH (Steinheim, Germany). Lipid standards such as PC (10:0-10:0), PE (10:0-10:0), PG (10:0-10:0), PI (8:0-8:0), LPC (13:0), LPE (14:0), LPG (14:0), LPI (13:0), SM (d18:1-12:0), and Cer (d18:1-12:0) were purchased from Avanti Polar Lipids (Alabaster, AL, USA), TG (11:1-11:1-11:1) and DG (8:0-8:0) were purchased from Larodan Fine Chemicals AB (Malmö, Sweden).

### 3.2. Preparation of the Extract of A. sessiliflorus Fruits (ASF)

*A. sessiliflorus* fruits were acquired in Jeongseon, Republic of Korea. A voucher specimen (NIHHS1501) was deposited at the Herbarium of the Department of Herbal Crop Research, National Institute of Horticultural and Herbal Science, Rural Development Administration, Eumseong, South Korea. The fruits of *A. sessiliflorus* were extracted with 50% aqueous fermented ethanol under reflux (70 °C) for 6 h, and extracted a second time with 50% aqueous fermented ethanol under reflux (70 °C) for 3 h. The extract was then filtered through a 5 μm filter. The supernatant was vacuum-concentrated under reduced pressure to attain 10–20 brix materials. Then, it was sterilized at 80–90 °C for 1 h. Finally, the ethanol extract of *A. sessiliflorus* fruits (ASF) was freeze-dried under reduced pressure (−30 °C, 100 mTorr) for 24 h. To establish the bulk-scale production of ASF, we optimized the manufacturing process based on an experiment scale.

### 3.3. Animal Study

Ten Wistar-Kyoto rats (WKY) and 50 male SHRs (aged 6 weeks), each weighing 180 ± 20 g, were procured from a commercial breeder (Saeronbio, Inc., Gyeonggi-Do, Korea). We obtained institutional review board approval for this study from the Korea Animal Medical Science Institute (No.15-KE-216). The rats were housed under controlled environmental conditions (12h light/dark cycle, temperature of approximately 23 ± 3 °C, and humidity of 55 ± 15%), with food and water available ad libitum throughout the experiments. Male WKYs were used as the normal control (G1), and the SHRs were randomly divided into five groups (n = 10 in each group), according to body weight—(G2) SHR (control); (G3) SHR-drug (a 4-week daily course of oral captopril at a dose of 100 mg/kg; (G4) SHR-ASF (a 4-week daily course of ASF at a dose of 200 mg/kg, p.o.); (G5) SHR-ASF (a 4-week daily course of ASF at a dose of 400 mg/kg, p.o.); and (G6) SHR-ASF (a 4-week daily course of ASF at a dose of 600 mg/kg, p.o.).

### 3.4. Sample Preparation

The rats were anesthetized using pentobarbital sodium, and blood samples were collected from the inferior vena cava in a vacutainer tube containing clot activator. The samples were kept at room temperature for 15–20 min, and centrifuged at 3000 rpm for 10 min to obtain the serum. The serum was aliquoted and stored in an ultra-low temperature freezer (−70 °C) for the analysis of the lipids.

### 3.5. Lipid Extraction

Each lipid standard, dissolved in chloroform/methanol (1:1 *v*/*v*), was stored at –20 °C and diluted to the desired concentration for use. In the lipid extraction of the mouse serum, we used a modified Bligh and Dyer method. Aliquots of 20 µL of serum were added to 990 µL of chloroform/methanol (1:2 *v*/*v*) and 10 µL of 1 µg/mL of lipid standards, including TG (11:1-11:1-11:1), DG (8:0-8:0), PC (10:0-10:0), PE (10:0-10:0), PG (10:0-10:0), PI (8:0-8:0), LPC (13:0), LPE (14:0), LPG (14:0), LPI (13:0), SM (d18:1-12:0), and Cer (d18:1-12:0) as internal standards (ISs). The sample was incubated for 10 min on ice. Then, 300 µL of chloroform and 390 µL of water were added and vortexed for 30 s. After centrifugation (6500× *g*, 5 min at 4 °C), the lower layer (organic phase) was transferred to a new tube. The sample was then dried using a SpeedVac concentrator, and was dissolved in 100 µL chloroform/methanol (1:1 *v*/*v*) for the LC/MS analysis.

### 3.6. UPLC–QTOF/MS Analysis

In the global lipid profiling, UPLC separation was performed using a Waters ACQUITY H-Class UPLC (Waters Corp., Milford, USA) with an ACQUITY BEH C18 column (2.1 mm × 100 mm, 1.7 µm). The temperatures of the column oven and sample tray were set to 40 °C and 4 °C, respectively. The mobile phases consisted of solvent A (acetonitrile–methanol–water mixture (19:19:2) + 20 mmol/L ammonium formate + 0.1% (*v*/*v*) formic acid) and solvent B (2-propanol + 20 mmol/L ammonium formate + 0.1% (*v*/*v*) formic acid). The gradient elution program was as follows—0–5 min, B 5%; 5–15 min, B 5–30%; 15–22 min, B 30–90%; 22–25 min, B 90%; 25–26 min, 90–5%; and 26–30 min, B 5%. The flow rate was 250 μL/min, and the injection volume was 2 μL for each run. The total run time was 30 min. Next, full mass scanning was performed using a Waters Xevo G2-S QTOF MS (Waters Corp., Milford, USA) operating in the positive and negative ion mode. The mass spectrometers performed alternative high- and low-energy scans, known as the MSE acquisition mode. The operating parameters were set as follows—cone voltage, 40 V; capillary, 3.0 kV; source temperature, 120 °C; desolvation temperature, 300 °C; cone gas flow, 30 L/h; and desolvation gas flow, 600 L/h. Accurate mass measurements were obtained by means of an automated calibration delivery system, which contained the internal reference [Leucine-enkephalin, m/z 556.276 (ESI+), m/z 554.262(ESI-)]. Data were collected between 100 and 1200 m/z.

### 3.7. UPLC–QqQ/MS Analysis

A 6490 Accurate-Mass Triple Quadrupole LC–MS coupled to a 1200 series HPLC system (Agilent Technologies, Wilmington, DE, USA) was used for the target lipid profiling. UPLC conditions, including the used column, mobile phases, gradient elution program, flow rate, and total run time, were equal to those of the UPLC–QTOF/MS analysis. The parameters of the operating source conditions were as follows—3500 V positive mode of capillary voltage, 3000 V negative mode of capillary voltage, sheath gas flow of 11 L/min (UHP nitrogen) at 200 °C, drying gas flow of 15 L/min at 150 °C, and nebulizer gas flow at 25 psi. The optimal multiple reaction monitoring (MRM) conditions were used to target various lipid species.

### 3.8. Data Processing and Statistical Analysis

All MS^E^ data obtained from UPLC—QTOF/MS were collected and processed within UNIFI 1.8 Beta (Waters Corp., Milford, USA). Data within UNIFI 1.8 was passed through the apex peak detection and alignment processing algorithms. This enabled related ion components to be grouped together and analyzed as a single entity. The intensity of each ion was normalized with respect to the total ion count, to generate a data matrix that consisted of the retention time (RT), m/z value, and the normalized peak area. Charged species, salt adducts, and fragments are all automatically aligned and grouped. Next, MRM data were obtained and processed using the Agilent Mass Hunter Workstation Data Acquisition software. The MRM data of the target lipids, including the m/z of precursor ions, m/z of product ions, and RT, were exported using Qualitative Analysis B.06.00 software (Agilent Technologies, Wilmington, DE, USA). An in-house database constructed using the Skyline software package (MacCoss Laboratory, University of Washington, Seattle, WA, USA) was then applied to determine the peak area of the assigned lipids from replicate raw data. The extracted areas of the lipid species were normalized to the appropriate IS. Statistical analyses, such as principal component analysis (PCA), sparse projection to latent structure discriminant analysis (sPLS–DA), and analysis of variance (ANOVA), were performed using the Metabo-Analyst website (http://www.metaboanalyst.ca).

## 4. Conclusions

Antihypertensive effect of the *A. sessiliflorus* fruits were attributed to the improvement of vascular relaxation and decrease in blood pressure in the hypertensive animal model [26]. Blood lipids are closely related to hypertension, and have recently been reported in view of the altered composition of lipids [4]. The conclusion of our study from this point of view, global lipid profiling by UPLC–QTOF/MS and target lipid profiling by UPLC–QqQ/MS were applied to characterize the lipid alterations of rat serum due to hypertension and treatment with a drug or ASF. Exact mass scanning using QTOF/MS was effective for identifying a total of 208 lipids from six pooled samples of the normal control, SHR, SHR + 100 mg/kg of Drug (captopril), SHR + ASF 200, 400, 600 mg/kg. PCA and sPLS–DA differentiated these six groups, and 120 lipids were selected as DRLs by ANOVA (*p* values < 0.05). Then, via the target profiling of 120 DRLs from individual samples of six groups and ANOVA (*p* values < 0.05), 67 lipids (38 TGs, 4 DGs, 17 PCs, 2 PEs, and 6 LPCs) were selected as vDRLs. In particular, among them, several lipid species such as TG (62:13), TG (60:13), PC (34:4), PC (36:5), and PC (38:2) were correlated with hypertension and several hypertension treatments. To our knowledge, this study was the first attempt to assess the levels of rat serum lipids altered by hypertension and treatment with a drug or ASF. Finally, these results demonstrated the correlation between hypertension and lipid metabolism in rat serum. The identified vDRLs also have the potential to support further detailed study of the metabolism of hypertension. However, further verification and quantification studies using larger independent sample sets are necessary to demonstrate the utility of these lipids as potential hypertension markers.

## Figures and Tables

**Table 1:** The list of validated differentially regulated lipids (vDRLs) of the six groups (normal control, SHR, SHR + 100 mg/kg of Drug, SHR + ASF 200, 400, 600 mg/kg) selected by the ANOVA test (*p* value < 0.05).

No.	Compounds	*p* Value	No.	Compounds	*p* Value
1	TG(14:0-14:0-14:0)	<0.001	35	TG(24:0-20:3-16:1)	0.005
2	TG(16:0-16:1-14:0)	<0.001	36	TG(22:6-20:4-18:2)	<0.001
3	TG(16:1-16:1-14:0)	0.006	37	TG(22:2-20:7-18:3)	<0.001
4	TG(18:0-16:1-14:0)	<0.001	38	TG(22:4-20:7-18:2)	<0.001
5	TG(18:1-16:1-14:0)	<0.001	39	DG(32:0)	0.019
6	TG(18:2-16:1-14:0)	0.004	40	DG(34:0)	0.039
7	TG(18:1-16:1-16:0)	0.033	41	DG(36:3)	0.012
8	TG(18:2-16:1-16:0)	0.006	42	DG(38:4)	0.003
9	TG(18:3-16:1-16:0)	0.024	43	LPC(14:0)	0.021
10	TG(20:0-18:1-16:0)	0.011	44	LPC(20:0)	<0.001
11	TG(20:1-18:1-16:0)	0.031	45	LPC(20:1)	0.001
12	TG(20:2-18:1-16:0)	0.009	46	LPC(20:2)	<0.001
13	TG(22:5-16:0-16:0)	0.001	47	LPC(20:3)	0.007
14	TG(20:3-18:2-16:0)	<0.001	48	LPC(22:0)	<0.001
15	TG(20:5-18:0-16:1)	0.001	49	PC(26:0)	<0.001
16	TG(20:4-18:2-16:0)	0.001	50	PC(30:0)	<0.001
17	TG(20:6-18:1-16:0)	0.029	51	PC(32:0)	<0.001
18	TG(20:5-18:2-16:1)	0.047	52	PC(32:2)	<0.001
19	TG(20:0-20:0-16:0)	0.006	53	PC(34:0)	0.003
20	TG(22:0-18:1-16:0)	0.003	54	PC(34:1)	<0.001
21	TG(20:2-18:1-18:0)	<0.001	55	PC(34:2)	<0.001
22	TG(20:2-18:1-18:1)	0.001	56	PC(34:3)	<0.001
23	TG(22:3-20:1-14:1)	0.001	57	PC(34:4)	<0.001
24	TG(20:5-20:1-16:0)	<0.001	58	PC(36:0)	<0.001
25	TG(20:3-18:1-18:3)	<0.001	59	PC(36:1)	<0.001
26	TG(20:4-18:2-18:2)	<0.001	60	PC(36:2)	<0.001
27	TG(20:6-20:1-16:2)	<0.001	61	PC(36:3)	<0.001
28	TG(20:0-20:2-18:0)	0.001	62	PC(36:5)	<0.001
29	TG(20:1-20:2-18:0)	0.009	63	PC(38:2)	<0.001
30	TG(20:1-20:6-18:0)	<0.001	64	PC(38:6)	<0.001
31	TG(20:3-20:4-18:1)	<0.001	65	PC(40:5)	<0.001
32	TG(20:4-20:5-18:1)	<0.001	66	PE(16:0)	<0.001
33	TG(20:3-20:5-18:3)	<0.001	67	PE(40:6)	<0.001
34	TG(22:0-20:1-18:2)	0.001			

## Supplementary Materials

Figure S1: PCA score plot of lipid profiles obtained from the six pooled samples of the normal control (G1), SHR (G2), SHR + Drug 100 mg/kg (G3), SHR + ASF 200 mg/kg (G4), SHR + ASF 400 mg/kg (G5), and SHR + ASF 600 mg/kg (G6). Table S1: Validation study of twelve lipid standards using UPLC–QTOF/MS and the LODs of each compound. Table S2: The list of various lipids analyzed from the six pooled samples (normal control, SHR, SHR + 100 mg/kg of Drug, SHR + ASF 200, 400, 600 mg/kg) of rat serum by UPLC–QTOF/MS. Table S3: The list of differentially regulated lipids (DRLs) of six pooled samples (normal control, SHR, SHR + 100 mg/kg of Drug, SHR + ASF 200, 400, 600 mg/kg) selected by ANOVA test (*p* value < 0.05).

## Abbreviations

ANOVA	Analysis of variance,
ASF	Extract of Acanthopanax sessiliflorus fruits,
CVD	cardiovascular disease,
Cer	Ceramide,
DG	Diacylglycerol,
DRL	Differentially regulated lipid,
LPA	Lysophosphatidic acid,
LPC	Lysophosphatidylcholine,
LPE	Lysophosphatidylethanolamine,
LPG	Lysophosphatidylglycerol,
LPI	Lysophosphatidylinositol,
LPS	Lysophosphatidylserine,
MRM	Multiple reaction monitoring,
MS	Mass spectrometry,
PA	Phosphatidic acid,
PC	Phosphatidylcholine,
PCA	Principal component analysis,
PE	Phosphatidylethanolamine,
PG	Phosphatidylglycerol,
PI	Phosphatidylinositol,
PS	Phosphatidylserine,
RSD	Relative standard deviation,
SHR	Spontaneously hypertensive rat,
SM	Sphingomyelin,
TG	Triacylglycerol,
UPLC	Ultra-performance liquid chromatography,
sPLS-DA	Sparse projection to latent structure discriminant analysis,
vDRLs	validated DRLs.
