# Supplementary material for: LC–MS-Based Lipidomic Analysis of Serum Samples from Spontaneously Hypertensive Rats Treated with an Extract of Acanthopanax sessiliflorus Fruits
Source: Molecules. 2020 Jul 17;25(14):3269. doi: 10.3390/molecules25143269 (PMC7397080; doi:10.3390/molecules25143269)
Supplement: Supplementary file 1 [file molecules-25-03269-s001.zip › Supplementary Figure S1.doc]

**Figure S1**. PCA score plot of lipid profiles obtained from six pooled samples of normal control (G1), SHR (G2), SHR + Drug 100 mg/kg (G3), SHR + ASF 200 mg/kg (G4), SHR + ASF 400 mg/kg (G5), and SHR + ASF 600 mg/kg (G6).
