# Supplementary material for: LC–MS-Based Lipidomic Analysis of Serum Samples from Spontaneously Hypertensive Rats Treated with an Extract of Acanthopanax sessiliflorus Fruits
Source: Molecules. 2020 Jul 17;25(14):3269. doi: 10.3390/molecules25143269 (PMC7397080; doi:10.3390/molecules25143269)
Supplement: Supplementary file 1 [file molecules-25-03269-s001.zip › Supplementary Table S1-S3.doc]

**Table S1**. Validation study of twelve lipid standards analysis using UPLC-QTOF/MS and the LODs of each compound.

| Lipid standards | RT  (min) | RSD (n=6) (%) | | Correlation  (*R*^2^) | Linear range (pg) | LOD^a)^ (pg) |
| --- | --- | --- | --- | --- | --- | --- |
|  |  | RT | Peak intensity |  |  |  |
| TG (11:1-11:1-11:1) | 3.96 | 0.5 | 9.1 | 0.9929 | 20 – 20000 | 20 |
| DG (8:0-8:0) | 1.46 | 0.4 | 5.1 | 0.9916 | 2 – 20000 | 2 |
| PC (10:0-10:0) | 2.15 | 0.5 | 5.8 | 0.9992 | 2 – 100000 | 2 |
| PE (10:0-10:0) | 1.75 | 0.5 | 9.1 | 0.9999 | 2 – 100000 | 2 |
| PG (10:0-10:0) | 1.39 | 0.7 | 9.9 | 0.9909 | 10 – 100000 | 10 |
| PI (8:0-8:0) | 1.11 | 0 | 6.9 | 0.9990 | 20 – 100000 | 20 |
| LPC (13:0) | 1.71 | 0.6 | 9.7 | 0.9963 | 20 – 10000 | 20 |
| LPE (14:0) | 1.46 | 0 | 8.3 | 0.9928 | 100 – 100000 | 100 |
| LPG (14:0) | 1.22 | 0 | 7.9 | 0.9973 | 10 – 200000 | 10 |
| LPI (13:0) | 1.12 | 0.7 | 8.9 | 0.9978 | 100 – 200000 | 100 |
| SM (d18:1-12:0) | 4.68 | 1.1 | 4.9 | 0.9967 | 1000 – 20000 | 1000 |
| Cer (d18:1-12:0) | 3.48 | 0.5 | 8.3 | 0.9930 | 200 – 10000 | 200 |

^a)^LOD: Limit of detection

**Table S2**. The list of various lipids analyzed from six pooled samples (normal control, SHR, SHR + 100 mg/kg of Drug, SHR + ASF 200, 400, 600 mg/kg) of rat serum by UPLC-QTOF/MS.

| No. | RT (min) | Compounds | Cn^a)^ | Un^b)^ | Molecular Formula | Expected Neutral Mass (Da) | Observed Neutral Mass (Da) | QTOF/MS  (ESI) (*m/z*) | Mass Accuracy (ppm) | Adducts |
| --- | --- | --- | --- | --- | --- | --- | --- | --- | --- | --- |
| 1 | 19.15 | TG(14:0-14:0-14:0) | 42 | 0 | C_45_H_86_O_6_ | 722.642 | 722.642 | 740.676 | 0.6 | +NH_4_ |
| 2 | 19.81 | TG(16:0-14:0-14:0) | 44 | 0 | C_47_H_90_O_6_ | 750.674 | 750.674 | 768.708 | 0.3 | +NH_4_ |
| 3 | 19.09 | TG(16:1-14:0-14:0) | 44 | 1 | C_47_H_88_O_6_ | 748.658 | 748.658 | 766.692 | 0.3 | +NH_4_ |
| 4 | 20.29 | TG(16:0-16:0-14:0) | 46 | 0 | C_49_H_94_O_6_ | 778.705 | 778.706 | 796.739 | 0.5 | +NH_4_ |
| 5 | 19.71 | TG(16:0-16:1-14:0) | 46 | 1 | C_49_H_92_O_6_ | 776.689 | 776.690 | 794.724 | 0.4 | +NH_4_ |
| 6 | 19.12 | TG(16:1-16:1-14:0) | 46 | 2 | C_49_H_90_O_6_ | 774.674 | 774.674 | 792.708 | 0.6 | +NH_4_ |
| 7 | 20.73 | TG(16:0-16:0-16:0) | 48 | 0 | C_51_H_98_O_6_ | 806.736 | 806.737 | 824.770 | 0.2 | +NH_4_ |
| 8 | 20.22 | TG(18:0-16:1-14:0) | 48 | 1 | C_51_H_96_O_6_ | 804.721 | 804.721 | 822.755 | 0.3 | +NH_4_ |
| 9 | 19.75 | TG(18:1-16:1-14:0) | 48 | 2 | C_51_H_94_O_6_ | 802.705 | 802.705 | 820.739 | 0.1 | +NH_4_ |
| 10 | 19.08 | TG(18:2-16:1-14:0) | 48 | 3 | C_51_H_92_O_6_ | 800.689 | 800.690 | 818.723 | 0.3 | +NH_4_ |
| 11 | 20.87 | TG(19:0-16:0-14:0) | 49 | 0 | C_52_H_100_O_6_ | 820.752 | 820.753 | 838.787 | 0.8 | +NH_4_ |
| 12 | 20.44 | TG(19:1-16:0-14:0) | 49 | 1 | C_52_H_98_O_6_ | 818.736 | 818.737 | 836.770 | 0.3 | +NH_4_ |
| 13 | 19.98 | TG(17:1-16:1-16:0) | 49 | 2 | C_52_H_96_O_6_ | 816.721 | 816.721 | 834.755 | 0.3 | +NH_4_ |
| 14 | 19.55 | TG(17:1-16:1-16:1) | 49 | 3 | C_52_H_94_O_6_ | 814.705 | 814.705 | 832.739 | 0.1 | +NH_4_ |
| 15 | 21.1 | TG(18:0-16:0-16:0) | 50 | 0 | C_53_H_102_O_6_ | 834.768 | 834.768 | 852.802 | 0.4 | +NH_4_ |
| 16 | 20.65 | TG(18:0-16:1-16:0) | 50 | 1 | C_53_H_100_O_6_ | 832.752 | 832.752 | 850.786 | 0.1 | +NH_4_ |
| 17 | 20.24 | TG(18:1-16:1-16:0) | 50 | 2 | C_53_H_98_O_6_ | 830.736 | 830.737 | 848.770 | 0.3 | +NH_4_ |
| 18 | 19.68 | TG(18:2-16:1-16:0) | 50 | 3 | C_53_H_96_O_6_ | 828.721 | 828.721 | 846.755 | 0.5 | +NH_4_ |
| 19 | 19.53 | TG(18:3-16:1-16:0) | 50 | 4 | C_53_H_94_O_6_ | 826.705 | 826.705 | 844.739 | 0.2 | +NH_4_ |
| 20 | 19.14 | TG(20:3-16:1-14:0) | 50 | 4 | C_53_H_94_O_6_ | 826.705 | 826.705 | 844.739 | 0.4 | +NH_4_ |
| 21 | 18.86 | TG(20:3-16:1-14:1) | 50 | 5 | C_53_H_92_O_6_ | 824.689 | 824.687 | 842.721 | 2.6 | +NH_4_ |
| 22 | 18.55 | TG(20:5-16:0-14:0) | 50 | 5 | C_53_H_92_O_6_ | 824.689 | 824.689 | 842.723 | 0.9 | +NH_4_ |
| 23 | 21.25 | TG(18:0-17:0-16:0) | 51 | 0 | C_54_H_104_O_6_ | 848.783 | 848.784 | 866.818 | 0.7 | +NH_4_ |
| 24 | 20.83 | TG(18:1-17:0-16:0) | 51 | 1 | C_54_H_102_O_6_ | 846.768 | 846.768 | 864.802 | 0.8 | +NH_4_ |
| 25 | 20.42 | TG(18:1-17:1-16:0) | 51 | 2 | C_54_H_100_O_6_ | 844.752 | 844.752 | 862.786 | 0.4 | +NH_4_ |
| 26 | 19.94 | TG(18:1-17:1-16:1) | 51 | 3 | C_54_H_98_O_6_ | 842.736 | 842.737 | 860.770 | 0.3 | +NH_4_ |
| 27 | 19.43 | TG(18:0-17:2-16:2) | 51 | 4 | C_54_H_96_O_6_ | 840.721 | 840.721 | 858.755 | 0.4 | +NH_4_ |
| 28 | 21.42 | TG(18:0-18:0-16:0) | 52 | 0 | C_55_H_106_O_6_ | 862.799 | 862.799 | 880.833 | 0.1 | +NH_4_ |
| 29 | 21.02 | TG(18:0-18:1-16:0) | 52 | 1 | C_55_H_104_O_6_ | 860.783 | 860.783 | 878.817 | 0 | +NH_4_ |
| 30 | 20.63 | TG(18:1-18:1-16:0) | 52 | 2 | C_55_H_102_O_6_ | 858.768 | 858.768 | 876.802 | 0.2 | +NH_4_ |
| 31 | 20.19 | TG(18:1-18:2-16:0) | 52 | 3 | C_55_H_100_O_6_ | 856.752 | 856.752 | 874.786 | 0.2 | +NH_4_ |
| 32 | 20.04 | TG(18:1-18:3-16:0) | 52 | 4 | C_55_H_98_O_6_ | 854.736 | 854.737 | 872.770 | 0.3 | +NH_4_ |
| 33 | 19.71 | TG(18:2-18:2-16:0) | 52 | 4 | C_55_H_98_O_6_ | 854.736 | 854.737 | 872.771 | 0.4 | +NH_4_ |
| 34 | 19.48 | TG(18:1-18:3-16:1) | 52 | 5 | C_55_H_96_O_6_ | 852.721 | 852.721 | 870.755 | 0.1 | +NH_4_ |
| 35 | 19.23 | TG(18:2-18:3-16:0) | 52 | 5 | C_55_H_96_O_6_ | 852.721 | 852.721 | 870.754 | 0.1 | +NH_4_ |
| 36 | 19.17 | TG(18:2-18:3-16:1) | 52 | 6 | C_55_H_94_O_6_ | 850.705 | 850.706 | 868.740 | 1.3 | +NH_4_ |
| 37 | 18.82 | TG(20:3-18:3-14:0) | 52 | 6 | C_55_H_94_O_6_ | 850.705 | 850.705 | 868.739 | 0 | +NH_4_ |
| 38 | 18.52 | TG(20:4-18:2-14:0) | 52 | 6 | C_55_H_94_O_6_ | 850.705 | 850.705 | 868.739 | 0.1 | +NH_4_ |
| 39 | 18.18 | TG(18:3-18:3-16:1) | 52 | 7 | C_55_H_92_O_6_ | 848.689 | 848.691 | 866.725 | 1.9 | +NH_4_ |
| 40 | 21.5 | TG(20:0-17:0-16:0) | 53 | 0 | C_56_H_108_O_6_ | 876.815 | 876.814 | 894.848 | 0.7 | +NH_4_ |
| 41 | 21.16 | TG(19:1-18:0-16:0) | 53 | 1 | C_56_H_106_O_6_ | 874.799 | 874.800 | 892.833 | 0.6 | +NH_4_ |
| 42 | 20.68 | TG(18:0-18:1-17:0) | 53 | 1 | C_56_H_106_O_6_ | 874.799 | 874.798 | 892.832 | 0.6 | +NH_4_ |
| 43 | 20.25 | TG(18:0-18:1-17:1) | 53 | 2 | C_56_H_104_O_6_ | 872.783 | 872.783 | 890.817 | 0.6 | +NH_4_ |
| 44 | 20.4 | TG(18:1-18:1-17:1) | 53 | 3 | C_56_H_102_O_6_ | 870.768 | 870.768 | 888.802 | 0.5 | +NH_4_ |
| 45 | 19.66 | TG(18:1-18:2-17:0) | 53 | 3 | C_56_H_102_O_6_ | 870.768 | 870.768 | 888.801 | 0.2 | +NH_4_ |
| 46 | 20.25 | TG(18:2-18:2-17:0) | 53 | 4 | C_56_H_100_O_6_ | 868.752 | 868.753 | 886.787 | 0.8 | +NH_4_ |
| 47 | 19.92 | TG(20:3-17:0-16:1) | 53 | 4 | C_56_H_100_O_6_ | 868.752 | 868.752 | 886.786 | 0.2 | +NH_4_ |
| 48 | 19.4 | TG(18:1-18:2-17:2) | 53 | 5 | C_56_H_98_O_6_ | 866.736 | 866.737 | 884.771 | 0.8 | +NH_4_ |
| 49 | 21.71 | TG(20:0-18:0-16:0) | 54 | 0 | C_57_H_110_O_6_ | 890.830 | 890.830 | 908.864 | 0 | +NH_4_ |
| 50 | 21.38 | TG(20:0-18:1-16:0) | 54 | 1 | C_57_H_108_O_6_ | 888.815 | 888.814 | 906.848 | 0.2 | +NH_4_ |
| 51 | 20.98 | TG(20:1-18:1-16:0) | 54 | 2 | C_57_H_106_O_6_ | 886.799 | 886.799 | 904.832 | 0.5 | +NH_4_ |
| 52 | 20.61 | TG(20:2-18:1-16:0) | 54 | 3 | C_57_H_104_O_6_ | 884.783 | 884.783 | 902.817 | 0 | +NH_4_ |
| 53 | 20.14 | TG(20:2-18:1-16:1) | 54 | 4 | C_57_H_102_O_6_ | 882.768 | 882.768 | 900.802 | 0.2 | +NH_4_ |
| 54 | 19.98 | TG(22:5-16:0-16:0) | 54 | 5 | C_57_H_100_O_6_ | 880.752 | 880.752 | 898.786 | 0.2 | +NH_4_ |
| 55 | 19.67 | TG(20:3-18:2-16:0) | 54 | 5 | C_57_H_100_O_6_ | 880.752 | 880.752 | 898.786 | 0.2 | +NH_4_ |
| 56 | 19.79 | TG(20:5-18:0-16:1) | 54 | 6 | C_57_H_98_O_6_ | 878.736 | 878.737 | 896.770 | 0.2 | +NH_4_ |
| 57 | 19.48 | TG(20:4-18:2-16:0) | 54 | 6 | C_57_H_98_O_6_ | 878.736 | 878.737 | 896.770 | 0.1 | +NH_4_ |
| 58 | 19.13 | TG(20:3-18:2-16:1) | 54 | 6 | C_57_H_98_O_6_ | 878.736 | 878.736 | 896.770 | 0.5 | +NH_4_ |
| 59 | 18.99 | TG(20:6-18:1-16:0) | 54 | 7 | C_57_H_96_O_6_ | 876.721 | 876.720 | 894.754 | 0.3 | +NH_4_ |
| 60 | 18.54 | TG(20:3-18:3-16:1) | 54 | 7 | C_57_H_96_O_6_ | 876.721 | 876.720 | 894.754 | 0.5 | +NH_4_ |
| 61 | 18.18 | TG(20:5-18:2-16:1) | 54 | 8 | C_57_H_94_O_6_ | 874.705 | 874.706 | 892.740 | 0.9 | +NH_4_ |
| 62 | 17.86 | TG(20:4-18:3-16:1) | 54 | 8 | C_57_H_94_O_6_ | 874.705 | 874.706 | 892.740 | 1 | +NH_4_ |
| 63 | 21.97 | TG(20:0-20:0-16:0) | 56 | 0 | C_59_H_114_O_6_ | 918.862 | 918.862 | 936.895 | 0.1 | +NH_4_ |
| 64 | 21.64 | TG(22:0-18:1-16:0) | 56 | 1 | C_59_H_112_O_6_ | 916.846 | 916.846 | 934.879 | 0.4 | +NH_4_ |
| 65 | 21.34 | TG(20:1-18:1-18:0) | 56 | 2 | C_59_H_110_O_6_ | 914.830 | 914.830 | 932.864 | 0 | +NH_4_ |
| 66 | 20.95 | TG(20:2-18:1-18:0) | 56 | 3 | C_59_H_108_O_6_ | 912.815 | 912.815 | 930.848 | 0 | +NH_4_ |
| 67 | 20.89 | TG(20:2-18:1-18:1) | 56 | 4 | C_59_H_106_O_6_ | 910.799 | 910.798 | 928.832 | 0.9 | +NH_4_ |
| 68 | 20.53 | TG(20:0-18:2-18:2) | 56 | 4 | C_59_H_106_O_6_ | 910.799 | 910.799 | 928.833 | 0.4 | +NH_4_ |
| 69 | 20.32 | TG(22:4-18:1-16:0) | 56 | 5 | C_59_H_104_O_6_ | 908.783 | 908.783 | 926.817 | 0.1 | +NH_4_ |
| 70 | 20.1 | TG(20:2-18:2-18:1) | 56 | 5 | C_59_H_104_O_6_ | 908.783 | 908.783 | 926.816 | 0.8 | +NH_4_ |
| 71 | 19.66 | TG(22:3-20:1-14:1) | 56 | 5 | C_59_H_104_O_6_ | 908.783 | 908.782 | 926.816 | 1.7 | +NH_4_ |
| 72 | 19.17 | TG(20:5-20:1-16:0) | 56 | 6 | C_59_H_102_O_6_ | 906.768 | 906.768 | 924.802 | 0.2 | +NH_4_ |
| 73 | 19.7 | TG(22:4-20:3-14:0) | 56 | 7 | C_59_H_100_O_6_ | 904.752 | 904.753 | 922.787 | 0.7 | +NH_4_ |
| 74 | 19.43 | TG(22:5-18:1-16:1) | 56 | 7 | C_59_H_100_O_6_ | 904.752 | 904.752 | 922.786 | 0.3 | +NH_4_ |
| 75 | 18.54 | TG(20:3-18:1-18:3) | 56 | 7 | C_59_H_100_O_6_ | 904.752 | 904.749 | 922.782 | 3.7 | +NH_4_ |
| 76 | 19.18 | TG(22:6-18:2-16:0) | 56 | 8 | C_59_H_98_O_6_ | 902.736 | 902.736 | 920.770 | 0.5 | +NH_4_ |
| 77 | 18.87 | TG(20:4-18:2-18:2) | 56 | 8 | C_59_H_98_O_6_ | 902.736 | 902.737 | 920.770 | 0.2 | +NH_4_ |
| 78 | 18.71 | TG(20:6-20:1-16:2) | 56 | 9 | C_59_H_96_O_6_ | 900.721 | 900.720 | 918.754 | 1 | +NH_4_ |
| 79 | 18.23 | TG(22:7-18:1-16:1) | 56 | 9 | C_59_H_96_O_6_ | 900.721 | 900.721 | 918.755 | 0.2 | +NH_4_ |
| 80 | 22.23 | TG(24:0-18:0-16:0) | 58 | 0 | C_61_H_118_O_6_ | 946.893 | 946.893 | 964.927 | 0.1 | +NH_4_ |
| 81 | 21.63 | TG(20:0-20:2-18:0) | 58 | 2 | C_61_H_114_O_6_ | 942.862 | 942.862 | 960.896 | 0.3 | +NH_4_ |
| 82 | 20.96 | TG(20:1-20:2-18:0) | 58 | 3 | C_61_H_112_O_6_ | 940.846 | 940.847 | 958.881 | 1.6 | +NH_4_ |
| 83 | 20.93 | TG(22:2-20:2-16:0) | 58 | 4 | C_61_H_110_O_6_ | 938.830 | 938.830 | 956.864 | 0.3 | +NH_4_ |
| 84 | 20.79 | TG(20:2-20:3-18:0) | 58 | 5 | C_61_H_108_O_6_ | 936.815 | 936.814 | 954.848 | 0.2 | +NH_4_ |
| 85 | 20.51 | TG(20:0-20:2-18:3) | 58 | 5 | C_61_H_108_O_6_ | 936.815 | 936.812 | 954.846 | 2.3 | +NH_4_ |
| 86 | 20.31 | TG(20:2-20:3-18:1) | 58 | 6 | C_61_H_106_O_6_ | 934.799 | 934.799 | 952.833 | 0.1 | +NH_4_ |
| 87 | 20.2 | TG(20:1-20:6-18:0) | 58 | 7 | C_61_H_104_O_6_ | 932.783 | 932.783 | 950.817 | 0 | +NH_4_ |
| 88 | 19.83 | TG(20:3-20:3-18:1) | 58 | 7 | C_61_H_104_O_6_ | 932.783 | 932.784 | 950.818 | 0.4 | +NH_4_ |
| 89 | 19.66 | TG(20:2-20:5-18:1) | 58 | 8 | C_61_H_102_O_6_ | 930.768 | 930.768 | 948.802 | 0.4 | +NH_4_ |
| 90 | 19.38 | TG(20:3-20:4-18:1) | 58 | 8 | C_61_H_102_O_6_ | 930.768 | 930.767 | 948.801 | 0.5 | +NH_4_ |
| 91 | 18.83 | TG(20:4-20:3-18:2) | 58 | 9 | C_61_H_100_O_6_ | 928.752 | 928.753 | 946.786 | 0.5 | +NH_4_ |
| 92 | 18.89 | TG(20:4-20:5-18:1) | 58 | 10 | C61H_98_O_6_ | 926.736 | 926.736 | 944.770 | 0 | +NH_4_ |
| 93 | 18.5 | TG(20:4-20:4-18:2) | 58 | 10 | C_61_H_98_O_6_ | 926.736 | 926.737 | 944.771 | 0.6 | +NH_4_ |
| 94 | 17.9 | TG(20:3-20:5-18:3) | 58 | 11 | C_61_H_96_O_6_ | 924.721 | 924.721 | 942.755 | 0.7 | +NH_4_ |
| 95 | 22.47 | TG(24:0-18:0-18:0) | 60 | 0 | C_63_H_122_O_6_ | 974.924 | 974.924 | 992.958 | 0.2 | +NH_4_ |
| 96 | 22.23 | TG(20:0-20:0-20:1) | 60 | 1 | C_63_H_120_O_6_ | 972.909 | 972.908 | 990.942 | 0.4 | +NH_4_ |
| 97 | 21.91 | TG(20:0-20:0-20:2) | 60 | 2 | C_63_H_118_O_6_ | 970.893 | 970.893 | 988.927 | 0.3 | +NH_4_ |
| 98 | 21.6 | TG(22:0-20:1-18:2) | 60 | 3 | C_63_H_116_O_6_ | 968.877 | 968.878 | 986.912 | 0.5 | +NH_4_ |
| 99 | 21.27 | TG(24:0-20:3-16:1) | 60 | 4 | C_63_H_114_O_6_ | 966.862 | 966.862 | 984.896 | 0.8 | +NH_4_ |
| 100 | 20.92 | TG(22:3-20:2-18:0) | 60 | 5 | C_63_H_112_O_6_ | 964.846 | 964.847 | 982.881 | 0.9 | +NH_4_ |
| 101 | 20.78 | TG(22:4-20:2-18:0) | 60 | 6 | C_63_H_110_O_6_ | 962.830 | 962.830 | 980.864 | 0.4 | +NH_4_ |
| 102 | 20.34 | TG(22:3-20:2-18:2) | 60 | 7 | C_63_H_108_O_6_ | 960.815 | 960.814 | 978.848 | 0.5 | +NH_4_ |
| 103 | 19.86 | TG(22:5-20:1-18:2) | 60 | 8 | C_63_H_106_O_6_ | 958.799 | 958.798 | 976.832 | 0.7 | +NH_4_ |
| 104 | 19.58 | TG(22:7-20:1-18:1) | 60 | 9 | C_63_H_104_O_6_ | 956.783 | 956.784 | 974.817 | 0.3 | +NH_4_ |
| 105 | 19.51 | TG(22:4-20:4-18:2) | 60 | 10 | C_63_H_102_O_6_ | 954.768 | 954.768 | 972.802 | 0.6 | +NH_4_ |
| 106 | 19.37 | TG(24:7-20:1-16:2) | 60 | 10 | C_63_H_102_O_6_ | 954.768 | 954.767 | 972.801 | 1 | +NH_4_ |
| 107 | 19.09 | TG(20:4-20:5-20:1) | 60 | 10 | C_63_H_102_O_6_ | 954.768 | 954.768 | 972.802 | 0.5 | +NH_4_ |
| 108 | 18.86 | TG(22:5-20:4-18:2) | 60 | 11 | C_63_H_100_O_6_ | 952.752 | 952.753 | 970.787 | 0.7 | +NH_4_ |
| 109 | 18.53 | TG(22:5-20:3-18:3) | 60 | 11 | C_63_H_100_O_6_ | 952.752 | 952.752 | 970.786 | 0.1 | +NH_4_ |
| 110 | 18.57 | TG(22:6-20:4-18:2) | 60 | 12 | C_63_H_98_O_6_ | 950.736 | 950.738 | 968.771 | 1.3 | +NH_4_ |
| 111 | 18.14 | TG(22:2-20:7-18:3) | 60 | 12 | C_63_H_98_O_6_ | 950.736 | 950.737 | 968.770 | 0.2 | +NH_4_ |
| 112 | 17.41 | TG(22:4-20:7-18:2) | 60 | 13 | C_63_H_96_O_6_ | 948.721 | 948.720 | 966.754 | 0.9 | +NH_4_ |
| 113 | 19.55 | TG(22:6-20:1-20:3) | 62 | 10 | C_65_H_106_O_6_ | 982.799 | 982.799 | 1000.833 | 0.2 | +NH_4_ |
| 114 | 19.32 | TG(22:3-20:7-20:1) | 62 | 11 | C_65_H_104_O_6_ | 980.783 | 980.783 | 998.817 | 0.1 | +NH_4_ |
| 115 | 19.02 | TG(22:9-20:2-20:0) | 62 | 11 | C_65_H_104_O_6_ | 980.783 | 980.783 | 998.817 | 0 | +NH_4_ |
| 116 | 18.78 | TG(22:8-22:2-18:2) | 62 | 12 | C_65_H_102_O_6_ | 978.768 | 978.768 | 996.802 | 0.2 | +NH_4_ |
| 117 | 18.17 | TG(22:7-20:2-20:3) | 62 | 12 | C_65_H_102_O_6_ | 978.768 | 978.768 | 996.802 | 0.1 | +NH_4_ |
| 118 | 18.51 | TG(22:5-20:7-20:1) | 62 | 13 | C_65_H_100_O_6_ | 976.752 | 976.753 | 994.786 | 0.6 | +NH_4_ |
| 119 | 18.14 | TG(24:7-22:5-16:1) | 62 | 13 | C_65_H_100_O_6_ | 976.752 | 976.754 | 994.788 | 2.2 | +NH_4_ |
| 120 | 17.72 | TG(22:6-20:4-20:4) | 62 | 14 | C_65_H_98_O_6_ | 974.736 | 974.736 | 992.770 | 0.1 | +NH_4_ |
| 121 | 12.96 | DG(32:0) | 32 | 0 | C_35_H_68_O_5_ | 568.507 | 568.506 | 591.495 | 0.8 | +Na |
| 122 | 15.24 | DG(34:0) | 34 | 0 | C_37_H_72_O_5_ | 596.538 | 596.539 | 619.528 | 0.9 | +Na |
| 123 | 13.37 | DG(34:1) | 34 | 1 | C_37_H_70_O_5_ | 594.522 | 594.523 | 617.512 | 0.6 | +Na |
| 124 | 11.37 | DG(34:2) | 34 | 2 | C_37_H_68_O_5_ | 592.507 | 592.507 | 615.496 | 0.1 | +Na |
| 125 | 17.28 | DG(36:0) | 36 | 0 | C_39_H_76_O_5_ | 624.569 | 624.570 | 647.559 | 0.3 | +Na |
| 126 | 14.16 | DG(36:1) | 36 | 1 | C_39_H_74_O_5_ | 622.554 | 622.551 | 640.585 | 3.6 | +Na |
| 127 | 13.32 | DG(36:2) | 36 | 2 | C_39_H_72_O_5_ | 620.538 | 620.536 | 643.526 | 2.4 | +Na |
| 128 | 11.28 | DG(36:3) | 36 | 2 | C_39_H_70_O_5_ | 618.522 | 618.522 | 641.511 | 0.9 | +Na |
| 129 | 9.13 | DG(36:4) | 36 | 4 | C_39_H_68_O_5_ | 616.507 | 616.507 | 639.497 | 1 | +Na |
| 130 | 12.38 | DG(38:4) | 38 | 4 | C_41_H_72_O_5_ | 644.538 | 644.538 | 667.527 | 0.6 | +Na |
| 131 | 9.08 | DG(38:7) | 38 | 7 | C_41_H_66_O_5_ | 638.491 | 638.491 | 639.499 | 0.3 | +H |
| 132 | 12.57 | DG(40:7) | 40 | 7 | C_43_H_70_O_5_ | 666.522 | 666.523 | 667.530 | 0.3 | +H |
| 133 | 3.85 | PC(26:0) | 26 | 0 | C_34_H_68_NO_8_P | 649.468 | 649.468 | 650.475 | 0.4 | +H |
| 134 | 7.71 | PC(30:0) | 30 | 0 | C_38_H_76_NO_8_P | 705.531 | 705.531 | 706.538 | 0.2 | +H |
| 135 | 10.29 | PC(32:0) | 32 | 0 | C_40_H_80_NO_8_P | 733.562 | 733.562 | 734.570 | 0.2 | +H |
| 136 | 7.74 | PC(32:1) | 32 | 1 | C_40_H_78_NO_8_P | 731.547 | 731.547 | 732.554 | 0.3 | +H |
| 137 | 5.7 | PC(32:2) | 32 | 2 | C_40_H_76_NO_8_P | 729.531 | 729.531 | 730.538 | 0.2 | +H |
| 138 | 12.69 | PC(34:0) | 34 | 0 | C_42_H_84_NO_8_P | 761.594 | 761.593 | 762.601 | 0.1 | +H |
| 139 | 10.29 | PC(34:1) | 34 | 1 | C_42_H_82_NO_8_P | 759.578 | 759.578 | 760.585 | 0.2 | +H |
| 140 | 8.11 | PC(34:2) | 34 | 2 | C_42_H_80_NO_8_P | 757.562 | 757.562 | 758.570 | 0.1 | +H |
| 141 | 6.56 | PC(34:3) | 34 | 3 | C_42_H_78_NO_8_P | 755.547 | 755.547 | 756.554 | 0.4 | +H |
| 142 | 5.12 | PC(34:4) | 34 | 4 | C_42_H_76_NO_8_P | 753.531 | 753.531 | 754.538 | 0.2 | +H |
| 143 | 14.85 | PC(36:0) | 36 | 0 | C_44_H_88_NO_8_P | 789.625 | 789.625 | 790.632 | 0.1 | +H |
| 144 | 12.62 | PC(36:1) | 36 | 1 | C_44_H_86_NO_8_P | 787.609 | 787.609 | 788.616 | 0 | +H |
| 145 | 10.06 | PC(36:2) | 36 | 2 | C_44_H_84_NO_8_P | 785.594 | 785.593 | 786.601 | 0.2 | +H |
| 146 | 8.50 | PC(36:3) | 36 | 3 | C_44_H_82_NO_8_P | 783.578 | 783.578 | 784.586 | 0.5 | +H |
| 147 | 6.01 | PC(36:4) | 36 | 4 | C_44_H_80_NO_8_P | 781.562 | 781.562 | 782.569 | 0.1 | +H |
| 148 | 5.17 | PC(36:5) | 36 | 5 | C_44_H_78_NO_8_P | 779.547 | 779.547 | 780.554 | 0.1 | +H |
| 149 | 4.52 | PC(36:6) | 36 | 6 | C_44_H_76_NO_8_P | 777.531 | 777.531 | 778.538 | 0.4 | +H |
| 150 | 14.7 | PC(38:1) | 38 | 1 | C_46_H_90_NO_8_P | 815.640 | 815.641 | 816.648 | 0.3 | +H |
| 151 | 12.96 | PC(38:2) | 38 | 2 | C_46_H_88_NO_8_P | 813.625 | 813.625 | 814.633 | 0.6 | +H |
| 152 | 10.29 | PC(38:3) | 38 | 3 | C_46_H_86_NO_8_P | 811.609 | 811.610 | 812.617 | 0.5 | +H |
| 153 | 9.30 | PC(38:4) | 38 | 4 | C_46_H_84_NO_8_P | 809.594 | 809.593 | 810.601 | 0.1 | +H |
| 154 | 8.15 | PC(38:5) | 38 | 5 | C_46_H_82_NO_8_P | 807.578 | 807.578 | 808.585 | 0.4 | +H |
| 155 | 6.39 | PC(38:6) | 38 | 6 | C_46_H_80_NO_8_P | 805.562 | 805.562 | 806.570 | 0.1 | +H |
| 156 | 4.52 | PC(38:7) | 38 | 7 | C_46_H_78_NO_8_P | 803.547 | 803.546 | 804.554 | 0.2 | +H |
| 157 | 15.04 | PC(40:2) | 40 | 2 | C_48_H_92_NO_8_P | 841.656 | 841.656 | 842.663 | 0.2 | +H |
| 158 | 12.65 | PC(40:3) | 40 | 3 | C_48_H_90_NO_8P_ | 839.640 | 839.641 | 840.648 | 0.1 | +H |
| 159 | 11.49 | PC(40:4) | 40 | 4 | C_48_H_88_NO_8_P | 837.625 | 837.625 | 838.632 | 0.2 | +H |
| 160 | 9.64 | PC(40:5) | 40 | 5 | C_48_H_86_NO_8_P | 835.609 | 835.609 | 836.617 | 0.1 | +H |
| 161 | 7.17 | PC(40:6) | 40 | 6 | C_48_H_84_NO_8_P | 833.594 | 833.594 | 834.601 | 0.2 | +H |
| 162 | 5.72 | PC(40:7) | 40 | 7 | C_48_H_82_NO_8_P | 831.578 | 831.578 | 832.585 | 0.1 | +H |
| 163 | 4.79 | PC(40:8) | 40 | 8 | C_48_H_80_NO_8_P | 829.562 | 829.562 | 830.569 | 0 | +H |
| 164 | 16.87 | PC(42:2) | 42 | 2 | C_50_H_96_NO_8_P | 869.687 | 869.687 | 870.694 | 0.7 | +H |
| 165 | 14.73 | PC(42:3) | 42 | 3 | C_50_H_94_NO_8_P | 867.672 | 867.673 | 868.680 | 1.6 | +H |
| 166 | 8.65 | PC(42:7) | 42 | 7 | C_50_H_86_NO_8_P | 859.609 | 859.609 | 860.617 | 0.3 | +H |
| 167 | 4.24 | PC(42:10) | 42 | 10 | C_50_H_80_NO_8_P | 853.562 | 853.562 | 854.570 | 0 | +H |
| 168 | 17.2 | PC(44:2) | 44 | 2 | C_52_H_100_NO_8_P | 897.719 | 897.720 | 898.727 | 1.2 | +H |
| 169 | 9.1 | PE(34:0) | 34 | 0 | C_39_H_78_NO_8_P | 719.547 | 719.547 | 720.554 | 0.2 | +H |
| 170 | 11.53 | PE(36:0) | 36 | 0 | C_41_H_82_NO_8_P | 747.578 | 747.578 | 748.586 | 0.5 | +H |
| 171 | 9.03 | PE(36:1) | 36 | 1 | C_41_H_80_NO_8_P | 745.562 | 745.563 | 746.570 | 0.4 | +H |
| 172 | 6.91 | PE(36:2) | 36 | 2 | C_41_H_78_NO_8_P | 743.547 | 743.547 | 744.554 | 0.2 | +H |
| 173 | 11.38 | PE(38:1) | 38 | 1 | C_43_H_84_NO_8_P | 773.594 | 773.594 | 774.601 | 0.1 | +H |
| 174 | 9.27 | PE(38:2) | 38 | 2 | C_43_H_82_NO_8_P | 771.578 | 771.578 | 772.585 | 0 | +H |
| 175 | 6.18 | PE(38:4) | 38 | 4 | C_43_H_78_NO_8_P | 767.547 | 767.547 | 768.554 | 0.2 | +H |
| 176 | 9.20 | PE(40:3) | 40 | 3 | C_45_H_84_NO_8_P | 797.594 | 797.595 | 798.602 | 1.4 | +H |
| 177 | 8.52 | PE(40:4) | 40 | 4 | C_45_H_82_NO_8_P | 795.578 | 795.578 | 796.585 | 0.1 | +H |
| 178 | 5.35 | PE(40:6) | 40 | 6 | C_45_H_78_NO_8_P | 791.547 | 791.547 | 792.555 | 1.1 | +H |
| 179 | 10.53 | PE(42:4) | 42 | 4 | C_47_H_86_NO_8_P | 823.609 | 823.610 | 824.617 | 0.9 | +H |
| 180 | 8.59 | PE(42:5) | 42 | 5 | C_47_H_84_NO_8_P | 821.594 | 821.594 | 822.602 | 1.1 | +H |
| 181 | 7.67 | PE(42:6) | 42 | 6 | C_47_H_82_NO_8_P | 819.578 | 819.578 | 820.585 | 0.1 | +H |
| 182 | 8.87 | PI(36:2) | 36 | 2 | C_45_H_83_O_13_P | 862.557 | 862.557 | 861.550 | 0.2 | -H |
| 183 | 5.84 | PI(36:4) | 36 | 4 | C_45_H_79_O_13_P | 858.525 | 858.526 | 857.519 | 0.7 | -H |
| 184 | 8.18 | PI(38:4) | 38 | 4 | C_47_H_83_O_13_P | 886.557 | 886.555 | 885.548 | 1.8 | -H |
| 185 | 1.34 | LPC(14:0) | 14 | 0 | C_22_H_46_NO_7_P | 467.301 | 467.301 | 468.308 | 0.6 | +H |
| 186 | 1.61 | LPC(16:0) | 16 | 0 | C_24_H_50_NO_7_P | 495.333 | 495.333 | 496.340 | 0.1 | +H |
| 187 | 1.37 | LPC(16:1) | 16 | 1 | C_24_H_48_NO_7_P | 493.317 | 493.317 | 494.324 | 0.1 | +H |
| 188 | 2.02 | LPC(18:0) | 18 | 0 | C_26_H_54_NO_7_P | 523.364 | 523.364 | 524.371 | 0.1 | +H |
| 189 | 1.62 | LPC(18:1) | 18 | 1 | C_26_H_52_NO_7_P | 521.348 | 521.348 | 522.355 | 0.1 | +H |
| 190 | 1.39 | LPC(18:2) | 18 | 2 | C_26_H_50_NO_7_P | 519.333 | 519.332 | 520.340 | 0.1 | +H |
| 191 | 1.24 | LPC(18:3) | 18 | 3 | C_26_H_48_NO_7_P | 517.317 | 517.317 | 518.324 | 0.4 | +H |
| 192 | 2.61 | LPC(20:0) | 20 | 0 | C_28_H_58_NO_7_P | 551.395 | 551.395 | 552.403 | 0.5 | +H |
| 193 | 2.02 | LPC(20:1) | 20 | 1 | C_28_H_56_NO_7_P | 549.379 | 549.380 | 550.387 | 0.1 | +H |
| 194 | 1.66 | LPC(20:2) | 20 | 2 | C_28_H_54_NO_7_P | 547.364 | 547.364 | 548.371 | 0.2 | +H |
| 195 | 1.43 | LPC(20:3) | 20 | 3 | C_28_H_52_NO_7_P | 545.348 | 545.348 | 546.356 | 0.4 | +H |
| 196 | 1.33 | LPC(20:4) | 20 | 4 | C_28_H_50_NO_7_P | 543.333 | 543.333 | 544.340 | 0.1 | +H |
| 197 | 1.18 | LPC(20:5) | 20 | 5 | C_28_H_48_NO_7_P | 541.317 | 541.317 | 542.325 | 0.7 | +H |
| 198 | 3.51 | LPC(22:0) | 22 | 0 | C_30_H_62_NO_7_P | 579.426 | 579.426 | 580.434 | 0.2 | +H |
| 199 | 1.52 | LPC(22:4) | 22 | 4 | C_30_H_54_NO_7_P | 571.364 | 571.364 | 572.371 | 0.4 | +H |
| 200 | 1.34 | LPC(22:5) | 22 | 5 | C_30_H_52_NO_7_P | 569.348 | 569.348 | 570.356 | 0.2 | +H |
| 201 | 1.27 | LPC(22:6) | 22 | 6 | C_30_H_50_NO_7_P | 567.333 | 567.333 | 568.340 | 0.2 | +H |
| 202 | 1.24 | LPE(16:0) | 16 | 0 | C_21_H_44_NO_7_P | 453.286 | 453.286 | 454.293 | 0.3 | +H |
| 203 | 1.34 | LPE(17:0) | 17 | 0 | C_22_H_46_NO_7_P | 467.301 | 467.301 | 468.308 | 0.9 | +H |
| 204 | 2.08 | LPE(18:0) | 18 | 0 | C_23_H_48_NO_7_P | 481.317 | 481.317 | 482.324 | 0.1 | +H |
| 205 | 1.48 | LPE(20:1) | 20 | 1 | C_25_H_50_NO_7_P | 507.333 | 507.333 | 508.340 | 0.9 | +H |
| 206 | 2.24 | LPE(22:0) | 22 | 0 | C_27_H_56_NO_7_P | 537.379 | 537.380 | 538.387 | 0.4 | +H |
| 207 | 1.8 | LPI(18:0) | 18 | 0 | C_27_H_53_O_12_P | 600.327 | 600.326 | 599.319 | 2 | -H |
| 208 | 1.23 | LPI(20:4) | 20 | 4 | C_29_H_49_O_12_P | 620.296 | 620.296 | 619.288 | 0.3 | -H |

^a)^ Cn: Total carbon chain length

^b)^ Un: Total degree of unsaturation

**Table S3**. The list of differentially regulated lipids (DRLs) of six pooled samples (normal control, SHR, SHR + 100 mg/kg of Drug, SHR + ASF 200, 400, 600 mg/kg) selected by ANOVA test (*p* value < 0.05).

| No. | Compounds | *p* value | No. | Compounds | *p* value |
| --- | --- | --- | --- | --- | --- |
| 1 | TG(14:0-14:0-14:0) | 0.022 | 61 | TG(20:3-20:3-18:1) | 0.044 |
| 2 | TG(16:0-16:1-14:0) | < 0.001 | 62 | TG(20:3-20:4-18:1) | < 0.001 |
| 3 | TG(16:1-16:1-14:0) | 0.001 | 63 | TG(20:4-20:5-18:1) | < 0.001 |
| 4 | TG(18:0-16:1-14:0) | < 0.001 | 64 | TG(20:3-20:5-18:3) | 0.013 |
| 5 | TG(18:1-16:1-14:0) | < 0.001 | 65 | TG(20:0-20:0-20:2) | < 0.001 |
| 6 | TG(18:2-16:1-14:0) | < 0.001 | 66 | TG(22:0-20:1-18:2) | < 0.001 |
| 7 | TG(19:1-16:0-14:0) | < 0.001 | 67 | TG(24:0-20:3-16:1) | < 0.001 |
| 8 | TG(17:1-16:1-16:1) | 0.013 | 68 | TG(22:3-20:2-18:2) | < 0.001 |
| 9 | TG(18:0-16:0-16:0) | < 0.001 | 69 | TG(22:4-20:4-18:2) | 0.028 |
| 10 | TG(18:0-16:1-16:0) | 0.044 | 70 | TG(22:5-20:4-18:2) | 0.042 |
| 11 | TG(18:1-16:1-16:0) | < 0.001 | 71 | TG(22:5-20:3-18:3) | < 0.001 |
| 12 | TG(18:2-16:1-16:0) | < 0.001 | 72 | TG(22:6-20:4-18:2) | 0.04 |
| 13 | TG(18:3-16:1-16:0) | < 0.001 | 73 | TG(22:2-20:7-18:3) | 0.008 |
| 14 | TG(20:3-16:1-14:0) | < 0.001 | 74 | TG(22:4-20:7-18:2) | 0.009 |
| 15 | TG(20:3-16:1-14:1) | 0.007 | 75 | TG(22:8-22:2-18:2) | 0.008 |
| 16 | TG(18:0-17:0-16:0) | 0.035 | 76 | TG(22:5-20:7-20:1) | < 0.001 |
| 17 | TG(18:1-17:0-16:0) | 0.030 | 77 | TG(24:7-22:5-16:1) | 0.033 |
| 18 | TG(18:1-17:1-16:0) | < 0.001 | 78 | TG(22:6-20:4-20:4) | < 0.001 |
| 19 | TG(18:1-17:1-16:1) | < 0.001 | 79 | DG(32:0) | < 0.001 |
| 20 | TG(18:0-17:2-16:2) | < 0.001 | 80 | DG(34:0) | < 0.001 |
| 21 | TG(18:0-18:0-16:0) | 0.001 | 81 | DG(34:2) | 0.003 |
| 22 | TG(18:0-18:1-16:0) | < 0.001 | 82 | DG(36:3) | < 0.001 |
| 23 | TG(18:1-18:1-16:0) | < 0.001 | 83 | DG(38:4) | 0.031 |
| 24 | TG(18:1-18:2-16:0) | < 0.001 | 84 | LPC(14:0) | 0.049 |
| 25 | TG(18:1-18:3-16:0) | < 0.001 | 85 | LPC(18:0) | 0.002 |
| 26 | TG(18:1-18:3-16:1) | < 0.001 | 86 | LPC(18:2) | < 0.001 |
| 27 | TG(18:2-18:3-16:0) | < 0.001 | 87 | LPC(20:0) | 0.001 |
| 28 | TG(18:2-18:3-16:1) | 0.027 | 88 | LPC(20:1) | 0.032 |
| 29 | TG(18:3-18:3-16:1) | < 0.001 | 89 | LPC(20:2) | < 0.001 |
| 30 | TG(19:1-18:0-16:0) | 0.002 | 90 | LPC(20:3) | 0.046 |
| 31 | TG(18:0-18:1-17:0) | 0.02 | 91 | LPC(22:0) | < 0.001 |
| 32 | TG(18:0-18:1-17:1) | < 0.001 | 92 | LPE(16:0) | 0.046 |
| 33 | TG(18:1-18:1-17:1) | < 0.001 | 93 | LPE(17:0) | 0.023 |
| 34 | TG(18:2-18:2-17:0) | < 0.001 | 94 | LPE(22:0) | 0.009 |
| 35 | TG(20:0-18:0-16:0) | 0.031 | 95 | PC(26:0) | 0.042 |
| 36 | TG(20:0-18:1-16:0) | < 0.001 | 96 | PC(30:0) | 0.008 |
| 37 | TG(20:1-18:1-16:0) | < 0.001 | 97 | PC(32:0) | 0.012 |
| 38 | TG(20:2-18:1-16:0) | < 0.001 | 98 | PC(32:2) | < 0.001 |
| 39 | TG(22:5-16:0-16:0) | < 0.001 | 99 | PC(34:0) | < 0.001 |
| 40 | TG(20:3-18:2-16:0) | < 0.001 | 100 | PC(34:1) | < 0.001 |
| 41 | TG(20:5-18:0-16:1) | < 0.001 | 101 | PC(34:2) | 0.043 |
| 42 | TG(20:4-18:2-16:0) | < 0.001 | 102 | PC(34:3) | 0.008 |
| 43 | TG(20:6-18:1-16:0) | < 0.001 | 103 | PC(34:4) | < 0.001 |
| 44 | TG(20:5-18:2-16:1) | < 0.001 | 104 | PC(36:0) | < 0.001 |
| 45 | TG(20:0-20:0-16:0) | 0.039 | 105 | PC(36:1) | < 0.001 |
| 46 | TG(22:0-18:1-16:0) | 0.014 | 106 | PC(36:2) | < 0.001 |
| 47 | TG(20:2-18:1-18:0) | < 0.001 | 107 | PC(36:3) | < 0.001 |
| 48 | TG(20:2-18:1-18:1) | 0.021 | 108 | PC(36:5) | 0.003 |
| 49 | TG(20:2-18:2-18:1) | 0.023 | 109 | PC(38:2) | 0.002 |
| 50 | TG(22:3-20:1-14:1) | < 0.001 | 110 | PC(38:3) | < 0.001 |
| 51 | TG(20:5-20:1-16:0) | < 0.001 | 111 | PC(38:6) | < 0.001 |
| 52 | TG(22:4-20:3-14:0) | < 0.001 | 112 | PC(38:7) | 0.044 |
| 53 | TG(22:5-18:1-16:1) | 0.007 | 113 | PC(40:2) | < 0.001 |
| 54 | TG(20:3-18:1-18:3) | 0.003 | 114 | PC(40:5) | < 0.001 |
| 55 | TG(20:4-18:2-18:2) | < 0.001 | 115 | PC(42:2) | < 0.001 |
| 56 | TG(20:6-20:1-16:2) | 0.013 | 116 | PC(42:7) | < 0.001 |
| 57 | TG(22:7-18:1-16:1) | < 0.001 | 117 | PC(44:2) | 0.022 |
| 58 | TG(20:0-20:2-18:0) | < 0.001 | 118 | PE(38:2) | 0.023 |
| 59 | TG(20:1-20:2-18:0) | < 0.001 | 119 | PE(40:6) | < 0.001 |
| 60 | TG(20:1-20:6-18:0) | 0.033 | 120 | PE(42:5) | 0.020 |
